# Assessing preconception health in Australia to support better outcomes in the first 2000 days – A critical need for building a core indicator framework

**DOI:** 10.1111/ajo.13815

**Published:** 2024-04-20

**Authors:** Asvini K. Subasinghe, Kirsten I. Black, Edwina Dorney, Jacqueline A. Boyle

**Affiliations:** ^1^ Department of Health Systems and Equity Eastern Health Clinical SchoolMonash University Melbourne Victoria Australia; ^2^ Faculty of Medicine and Health The University of Sydney Sydney New South Wales Australia; ^3^ Sydney Local Health District New South Wales Australia

**Keywords:** attitudes, health knowledge, intention, maternal health, practice, preconception care, pregnancy, prevention & control, public health, social determinants of health, women's health

## Abstract

In 2021, the Preconception Health Network Australia co‐developed preconception health core indicators identified as critical to ensuring optimal maternal and child outcomes following conception. We conducted an audit of perinatal databases across each state and territory to identify whether preconception core indicator data were available. Seven health domains co‐developed by the Preconception Health Network were mapped against the data collected in the perinatal databases. Indicator data were lacking across all seven health domains, with data missing for social determinants of health indicators. Better data linkage and developing a national evidence‐based framework would allow ongoing monitoring of women's preconception health nationally.

## INTRODUCTION

Health, behaviours and wellbeing prior to pregnancy, and between pregnancies, can impact on the quality of one's pregnancy and the future health of women and their offspring.^1^, ^2^ The importance of optimising preconception health is highlighted in clinical guidelines and policies published nationally and internationally.^3^ A preconception population is variably defined, but most frameworks include a life‐course approach, a public health approach for those of reproductive age and an individual approach for those actively intending pregnancy or the health of those prior to a current pregnancy, the latter of which we are using in this paper.^1^, ^4^ In order to understand the status of Australia's preconception health, there is a need to identify, develop and monitor indicators of preconception health.^2^

**Figure 1:**
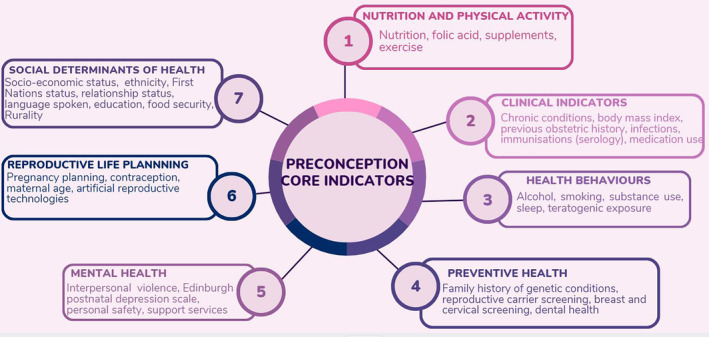
Preconception health core indicators developed across seven health domains.

The Preconception Health Network Australia (PCHN), auspiced by the Centre of Research Excellence in Health in Preconception and Pregnancy (CRE HiPP) identified data and monitoring of preconception health and care as one of the critical priority areas for attention in Australia.^5^ As such, the PCHN has co‐developed a set of core indicators suitable to monitor preconception health and care in Australia.^5^, ^6^ This was achieved by bringing together researchers, clinicians, policy experts, and community representatives to identify a set of core indicators across seven health domains^6^ using a modified Delphi and Nominal Group Technique approach (Fig. 1). These domains demonstrate the complex interactions of biomedical, behavioural, social determinants of health, and emotional wellbeing factors as key components of preconception heath and care.

Monitoring readily accessible pregnancy data enables the evaluation of disparities in preconception health, demonstrated by research from the UK.^2^ In order to plan for a similar process in Australia, we therefore aimed to assess national and state‐based pregnancy data sets in Australia and to map the data to the core indicators established by the PCHN.

## MATERIALS AND METHODS

Using these core indicators, we conducted an audit of all the Australian state and territory‐based perinatal databases, and the Maternity Information Matrix (MIM) (a national database derived from the state databases) to assess to what extent the indicators were captured in routinely collected data. We sent our list of preconception health core indicators to data custodians for each perinatal database to check whether the data were available. Data custodians provided Yes/No responses, against each of the core indicators on our list for the perinatal databases from the Australian Capital Territory, New South Wales, South Australia and Western Australia. We were directed to consult the data variable dictionaries available for the perinatal databases for Victoria, Northern Territory, Tasmania and Queensland. These databases were reviewed in the absence of dedicated preconception health databases. Data custodians also provided availability from other data sources such as population health surveys for reference, but these datasets were not linked to perinatal databases.

## RESULTS

Data presented are from state and territory based perinatal databases as data custodians from the MIM informed us that perinatal databases for each jurisdiction have the most current data dictionaries. Information regarding chronic conditions, physical health, previous obstetric history, alcohol and smoking, screening for interpersonal violence, age, and ethnic background were mostly available across all states. However, data were not complete for some screening variables. For example, data were collected on whether or not a screening for interpersonal violence was conducted, but the result of that screening was not collected. Additionally, data were lacking nationwide for diet and supplements and missing for exercise, sleep, reproductive life planning, and the entire preventive health domain (Table 1). Importantly, no state data were available for a family history of genetic conditions, or previous pregnancy genetic conditions, reproductive carrier screening, cervical/breast screening, dental health, contraception use, language spoken, personal safety, employment or education level.

**Table 1:** Availability of preconception health core indicator data across state and territory‐based perinatal databases

Health domain	State/territory							
	Victoria	NSW	ACT	Tas	WA	QLD	NT	SA
Nutrition and physical activity								
Fruit and veg			O					
Folic acid			O	O				
Anaemia			O					
Supplements			O	O				
Clinical indicators								
Hypertension	O	O	O	O	O			O
Diabetes	O	O	O	O	O			O
Epilepsy			O	O				O
Asthma			O					O
Polycystic ovarian syndrome			O					O
Cancer			O					O
Autoimmune disease			O					
Physical health								
Body mass index	O	O	O	O	O	O		O
Previous obstetric history	O							
Parity	O	O	O		O		O	O
Preterm birth			O					
Gestational diabetes	O							O
Pre‐eclampsia								O
Caesarean section	O		O	O	O	O		O
Abortions	O		O	O	O	O		O
Preterm labour	O		O	O	O	O		O
Infections								
Sexually transmitted infections	O							
Immunisations	O			O	O	O		O
Health behaviours								
Alcohol	O			O	O	O	O	O
Smoking and vaping	O			O	O	O	O	O
Substance use				O	O	O		O
Sleep								
Environmental exposures								
Preventive health								
Genetics, family history, carrier screening, cervical/breast screening, dental								
Mental and emotional health								
Mental health	O			O				
Edinburgh postnatal depression scale score	O				O	O		
Interpersonal violence/family violence screening status	O			O	O	O		
Personal safety		O						
Supports								
Reproductive life planning								
Pregnancy planning, contraception								
Maternal age	O	O	O	O	O		O	O
Assisted reproductive technology								
Gestational age at booking	O	O		O	O		O	O
Social determinants of health								
Food security								
Poverty								
Education								
Ethnicity		O			O			
Rurality/postcode	O	O		O		O	O	O
Housing								
Coordination of all care with primary care								
Country of birth		O	O					
Language spoken								
First Nations status	O	O		O		O	O	O

‘O’ depicts data being available in a state/territory perinatal dataset.

ACT, Australian Capital Territory; NSW, New South Wales; SA, South Australia; WA, Western Australia; VIC, Victoria; NT Northern Territory; TAS, Tasmania; QLD, Queensland.

## DISCUSSION

These findings highlight that while some indicators of preconception health are being collected in state and national perinatal data, some important gaps remain across all seven domains. There are differences between how preconception indicator data are collected by states and territories, such as body mass index and ethnicity, and for some other key indicators (eg social determinants of health) no data are collected. Mapping preconception health indicators using the perinatal databases is therefore currently not possible. Yet it is critical that we understand the impacts of preconception policy, health and social care and research on pregnancy outcomes.

The PCHN has recommended that Australia builds a national evidence‐based framework to capture and report preconception health and care indicator data to enable longitudinal monitoring of equity, health behaviours, healthcare and health outcomes and to enable benchmarking against preconception health frameworks internationally.^2^ This would require a multi‐pronged approach. Firstly, based on clinical experience, relevant data for a number of these indicators such as rubella serology, medication and supplement use preconception, need for an interpreter, are collected in many hospital pregnancy records but are not transferred to the state or national perinatal databases. The reasons for this are varied but include a focus on collecting specified pregnancy indicators and that current practice is to send only pre‐formatted/pre‐determined data extracts from hospital records to state databases. We could potentially conduct an audit of existing maternity databases for content, and identify which fields are pre‐formatted, and which fields are mandatory and map to our indicators. Additionally, for the free text we could employ large language models to read these data as there is potential to develop a map that can be applied across different electronic medical records platforms that would assist in collecting data on preconception health indicators. There are a number of approaches to standardisation of the structure and content of data from disparate health systems that could potentially enable data collection and efficient and reliable analyses. Given the different systems used in pregnancy care in hospitals across Australia, a comprehensive national system would take time to develop. However, piloting and testing these mechanisms and collaboratively generating evidence that promotes better health decisions and better care can enable robust monitoring of future preconception health. Finally, national preconception health surveys could be developed and administered to capture missing data on critical preconception health core indicators. These recommendations are aligned with the Preconception Health Network's recommendations for the Senate Inquiry into universal access to sexual and reproductive health.^7^

Producing the first national report card for the state of preconception health for women in Australia has the potential to identify priority preconception health indicators for ongoing monitoring at a national level and targeting interventions with the aim of reducing adverse pregnancy outcomes for women across Australia, particularly those from priority groups at greatest risk of poor outcomes.
